# The Training of the General Practitioner for Obstetrics

**Published:** 1917-12

**Authors:** James E. King

**Affiliations:** Buffalo, N. Y.


### THE TRAINING OF THE GENERAL PRACTITIONER FOR OBSTETRICS.*

**By James E. King, M.D., F.A.C.S., Buffalo, N. Y.**

There is no more interesting chapter in medicine than that of obstetrics. Its development as a science and art has been rather slow. It is only within a comparatively short time that it has occupied a dignified place in medicine. Through all ages and among every people, it has been a woman's business to attend women in labor, and such duties were relegated to the lowest and most ignorant. No man was ever permitted in the presence of a woman in labor except in the most difficult and complicated cases, when the aid of priests or men of mystic powers was sought. History records that in 1522 Wr. Wertt, of Hamburg, dressed in woman's clothes, for the praiseworthy purpose of attending and studying a case of labor. Unfortunately, he was discovered, and paid dearly for his attempt. He was burned alive. One hundred years later a Dr. Willoughby, wishing to assist his daughter in a difficult labor, was obliged to crawl into the darkened room upon his hands and knees. Some years later it was recognized as proper for a physician to be called in difficult and complicated cases, but only as a last resort. Version and embryotomy were his chief expedients and death frequently followed in his wake.

The first step taken in the improvement in the care of normal cases was the establishment of French and German schools for midwives. A better class of women then took up the work, and in general it was better done. The physician was still barred from the normal case, not only be reason of the prejudice of the laity, but also because attendance upon a normal case was regarded by the profession as beneath the dignity and calling of the physician. Gradually, however, physicians displaced the midwife, to a very limited extent, in normal cases, but these men were not the best of the profession. They were held by their confers in the same contempt as were the men of more recent years who devoted themselves to the treatment of venereal disease. The teaching of obstetrics in the medical schools was entirely by didactic lecture, and whatever clinical experience the student acquired was that gained in attendance upon such cases with his preceptor. Such opportunities afforded little in practical instruction, for it was considered an unpardonable sin to in any way expose a patient at any stage of labor. All operations and deliveries were conducted under the protecting sheet, and woe betide the attendant who, by accident or design, exposed even for a moment his patient! Under such conditions but little could be expected from the recent graduate in medicine, and normal cases were unquestionably much better cared for by the well trained and experienced midwife.

The University of Buffalo wrote a page in the history of obstetric teaching, when in 1850, Dr. James P. White, then Professor of Obstetrics in that institution, demonstrated a normal labor at the hospital to a class of students. This brought down upon his devoted head the most harsh criticism, and in the bitter controversy which followed the laity and the profession took part. The daily press was caustic in its criticisc, and the medical journals which did not condemn, attempted to excuse his action by timidly setting forth the possible advantages of such methods of teaching. This culminated in a libed suit which attracted wide-spread attention. And this is in the memory of men alive today! Since that time the laity has recognized the necessity and advantages of better obstetrics, and has contributed liberally to founding and endowing institutions throughout the country where the poor may receive better care during confinement, and, at the same time, where students and graduates of medicine may receive better instruction. The practice of obstetrics has thus not only been elevated to an honorable position in the profession, but the laity has come to recognize it as a highly specialized branch of medicine.

Although the great improvement in the practice and teaching of this branch of medicine is generally recognized, there is still in certain quarters unrest in the profession. We ask and are asked whether the mean average of obstetric practice is at that level which we have a right to expect it to be by reason of our advanced obstetric knowledge and the better facilities and methods of imparting that knowledge to studends and practitioners. Even with the most casual survey of the situation, we have it painfully and forcefully borne upon us, that in general, obstetric practice does not measure up to what we are justified in regarding as present-day ideals.

Recognizing this fact the inquiring mind at once addresses itself to determining the reasons why obstetrics as actually practiced today, does not approach more nearly what we have a right to consider as an attainable ideal. This question has been variously answered, and various remedies have been proposed. For an intelligent consideration of the subject, one must first clearly recognize that no one factor explains entirely why obstetric practice falls short of our ideal. We may ask ourselves if the present obstetric training received by the average medical student is of such a kind as to justify our expecting better practice, or is this training all that can be desired and does the fault lie in the men themselves? Before arguing these questions, let us take a brief and general survey of obstetric practice as we find it.

In all cities with a large foreign population obstetric practice is about evenly divided between the midwife and general practitioner. In such cities a small number of the better class employ the specialist and a small number of the poorer class avail themselves of free hospital service. Although the number cared for by the specialist and hospitals is by comparison insignificant, the well-to-do and the very poor in such cities have the best of their service if they choose. The overwhelming majority of women, however, are confined by the midwife or general practitioner. Leaving out of consideration for the present the cases attended by midwifes, we may consider briefly the general practitioner and his work in this field.

Broadly speaking, practitioners may be divided into two groups: those who do, and those who do not, like obstetrics. Those who do not, engage in the work for one or both of two reasons, because of the income, or because their clientele demands it and if they refuse, it results in the loss of the family. From such men good obstetrics cannot be expected. Regarding it as a necessary evil, their work is hurried and unscientific. From the second class of practitioner we may look for better work. To them obstetrics is congenial. Their interest not only prompts good work, but is an incentive to study and to perfect themselves.

Let us turn now for a moment to the attitude of the laity. Formerly the influence of midwife practice brought the physician who attended normal cases to the midwife's level. The compensation the physician received for such work was but little more than that of the midwife. It is a curious fact that, although in the eyes of the laity, the physician has succeeded in raising himself from the midwife's level, the vast majority are still attending confinements for little more than the midwife's fee. The women of today, expect much more from the physician, and among the more intelligent class, his watchful attention is demanded during pregnancy, and his asepsis and technique during labor are critically observed. These are hopeful signs, and with encouragement and further education by the profession, these women will one day realize that for such service as they expect, a reasonable and proper fee must be paid.

Now, having briefly presented certain angles of obstetric practice, it is proper to inquire what the shortcomings of the profession are that merit criticism, and to decide how far such faults may be attributed to deficient training. Pregnancy terminating in a normal labor is fundamentally a natural physiologic process. Largely, however, as a result of the evolution of man and his higher civilization, pregnancy and labor approach closely the border line of the abnormal. For this reason a watchful vigilance during pregnancy is necessary. Does the profession in general sufficiently appreciate this fact? Many do, but a greater number slight the prophylactic care of pregnancy. This neglect of physicians may, to a certain extent, be attributed to the faulty training of many schools which fail to emphasize by clinical teaching its importance. There are physicians, however, even knowing its importance, who prefer to place reliance in the blind faith that nature will not stray over the border line of the normal. From such men we can hope for better things only when an educated public demand of them that which they do not willingly give. There are indications that that day is drawing near.

Our next criticism may be offered in the failure in proper aseptic technique. Labor is to be considered as a surgical operation and as such demands surgical asepsis. Errors in asepsis during labor are not always due to improper training, but because the physician's duties during labor in the home are often many sided, and a perfect technique cannot be maintained. Some physicians still confine women without gloves. No excuse can be offered for such men. Training cannot be held responsible for this, and these men will continue, and will be followed by others, until our educated public insists on better things. The most common defiiciency, however, all consultants will agree, lies in diagnosis. The blame for this can be laid almost entirely to faulty training. Instructors fail to insist upon the paramount importance of diagnosis, and to irrevocably impress upon the student that diagnosis is the indispensible requisite for good obstetrics. It is too true that many physicians trust hopefully that the case in hand is normal, and only in the presence of unmistakeable indications of trouble, is a diagnosis attempted. Such men are still, and will continue to be, midwives. In some instances, the fault is in the man and not in his training. Unfortunately, however, in many schools, the clinical material is not sufficient, and the teaching staff not painstaking enough in this direction. The cure is obvious.

More deplorable in its effects upon the patient are unjustified efforts to terminate labor quickly. This is a common offense, and has been characterized as meddlesome midwifery. Such ill-advised treatment can be attributed in some cases, to ignorance, but more often it is done simply because it suits the convenienee of the physician. Such men are willing to trust most complacently in nature for the safe progress of pregnancy, and trusting also that she has pre-arranged a normal presentation and position, consider diagnosis superflous. But these same men, with curious inconsistency, after nature has partially dilated a cervix, lost all further confidence, and take the matter entirely out of her hands!

It is in the use of forceps that such offenses are usually committed. Dragging the head through an incompletely dilated cervix leaves in its train the conditions which the gynecologist must later rectify. No amount of training will perhaps, ever entirely correct this. There are too many factors that enter in as causes of this abuse. If, in the training of the student, more emphasis could be laid upon the abuse of forceps, and if the unfortunate results upon both mother and child, which often attend their use, could be more often demonstrated, some progress might be made. The impression usually left with the student is the one made in the clinic by an expert who easily applies and delivers with forceps.

To the gynecologist, it would seem that training might be improved in another direction. Birth-canal injuries of greater or lesser extent will occur in a certain proportion of labors. These injuries are often belittled, and their repair hastily and improperly done. The physician often feels that when a tear occurs he is open to criticism. In order to minimize the injury, he passes two sutures through the perineal body, with as little ado as possible. A careful inspection of these injuries would show how impossible it is to correctly repair them in this way. If men were taught that in such injuries there are definite structures which must be properly adjusted to each other, and that to do this proper position, good light, and complete inspection are necessary, the gynecologist would soon find his income diminishing.

Those who devote themselves to the training of students recognize the difficulty of teaching obstetrics as it properly should be taught, in connection with the curriculum of the medical school. The element of time is such an important factor that certain phases of clinical obstetric teaching are impossible. The ideal method would, of course, be for a student to follow a patient in the late weeks of pregnancy, and then to have every feature of the labor carefully demonstrated by a competent obstructor. If it were possible to study two or three cases in this way, it would be of infinitely greater value than the present system of simply requiring attendance at a certain number of confinements. Such methods as adopted by our present day schools, manifestly cannot attain this teaching ideal, and students who enter practice with only such training are the ones who recruit the ranks of those doing poor obstetrics. Those graduates who do not have the advantage of an interneship in a hospital where a good maternity service is maintained, should avail themselves of the opportunities offered by the many large obstetric clinics for post-graduate work.

It is impossible in such a paper to consider all the shortcomings of obstetric teaching and the remedies. One has only to keep in mind, however, the marvelous improvement in teaching in the last twenty-five years, to have confidence that improvement will still go on, and that the unsolved problems of obstetrics teaching to-day will find their answer to-morrow.

The writer does not wish to close without paying tribute to the scientific and conscientious obstetric work of many general practitioners. Such men are often found in small communities, and their methods and results could do credit to the best. Those who, in spite of training, will not do good work, the writer is satisfied to leave to the tender mercies of the public. When women are further educated to the requirements of safe and good obstetrics, such men will be speedily and surely eliminated.—New York State Journal of Medicine.

